# Effect of gibberellic acid on photosynthesis and oxidative stress response in maize under weak light conditions

**DOI:** 10.3389/fpls.2023.1128780

**Published:** 2023-02-16

**Authors:** Jianjun Fu, Linlin Li, Shuang Wang, Na Yu, Hong Shan, Zhensheng Shi, Fenghai Li, Xuemei Zhong

**Affiliations:** ^1^ Special Corn Institute, Shenyang Agricultural University, Shenyang, China; ^2^ Liaoning Dongya Seed Co., Ltd., Shenyang, China

**Keywords:** maize, low light stress, GA3, barren stalk, photosynthesis

## Abstract

Gibberellin (GA) is an important endogenous hormone involved in plant responses to abiotic stresses. Experiments were conducted at the Research and Education Center of Agronomy, Shenyang Agricultural University (Shenyang, China) in 2021.We used a pair of near-isogenic inbred maize lines comprising, SN98A (light-sensitive inbred line) and SN98B (light-insensitive inbred line) to study the effects of exogenous gibberellin A3 (GA_3_) application on different light-sensitive inbred lines under weak light conditions. The concentration of GA_3_ was selected as 20, 40 and 60 mg L^-1^. After shade treatment, the photosynthetic physiological indexes of SN98A were always lower than SN98B, and the net photosynthetic rate of SN98A was 10.12% lower than SN98B on the 20th day after shade treatment. GA_3_ treatments significantly reduced the barren stalk ratios in SN98A and improved its seed setting rates by increasing the net photosynthetic rate (Pn), transpiration rate (Tr), stomatal conductance (Gs), photosynthetic pigment contents, photochemical efficiency of photosystem II (PS II) (Fv/Fm), photochemical quenching coefficient (qP), effective quantum yield of PSII photochemistry (Φ_PSII_), and antioxidant enzyme activities, where the most effective treatment was 60 mg L^–1^GA_3_. Compared with CK group, the seed setting rate increased by 33.87%. GA_3_ treatment also regulated the metabolism of reactive oxygen species (ROS) and reduced the superoxide anion ( ${\text{O}}_{2}^{-}$ ) production rate, H_2_O_2_ content, and malondialdehyde content. The superoxide anion ( ${\text{O}}_{2}^{-}$ ) production rate, H_2_O_2_ content and malondialdehyde content of SN98A sprayed with 60 mg L^-1^ GA_3_ decreased by 17.32%,10.44% and 50.33% compared with CK group, respectively. Compared with the control, GA_3_ treatment significantly (*P* < 0.05) increased the expression levels of *APX* and *GR* in SN98A, and *APX*, *Fe-SOD*, and *GR* in SN98B. Weak light stress decreased the expression of *GA20ox2*, which was related to gibberellin synthesis, and the endogenous gibberellin synthesis of SN98A. Weak light stress accelerated leaf senescence, and exogenous GA_3_ application inhibited the ROS levels in the leaves and maintained normal physiological functions in the leaves. These results indicate that exogenous GA_3_ enhances the adaptability of plants to low light stress by regulating photosynthesis, ROS metabolism and protection mechanisms, as well as the expression of key genes, which may be an economical and environmentally friendly method to solve the low light stress problem in maize production.

## Introduction

Under suitable cultivation conditions, the productivity of different crops is strongly related to the amount of light radiation intercepted in the crop canopy (Slattery and Ort, 2021), where excessive or insufficient amounts of light energy will have adverse effects on photosynthesis by crops (Ferrante and Mariani, 2018). Maize (*Zea mays* L.) is a light-loving and light-sensitive crop (Wang et al., 2016; Xue et al., 2019), but due to frequent extreme weather events in recent years, maize plants have experienced continuous low-temperature and rainy weather in the booting stage. These conditions can severely affect ear development and grain formation in maize, thereby resulting in large areas which high proportions of hollow straw and severe bald tip in some varieties (Huang et al., 2022), which are extremely unfavorable for agricultural production. Light is necessary for photosynthesis and it is the basis of plant life. Insufficient light will lead to decreases in the net photosynthetic rate (Pn) and partial chlorophyll fluorescence parameters in leaves (Qu et al., 2017; Wegrzyn and Mazur, 2020; Feng et al., 2021; Zahra et al., 2022). During this process, plant leaf cells undergo complex changes in physiological processes and cell metabolism, such as chloroplast decomposition, loss of photosynthetic activity, decomposition of chlorophyll and macromolecular compounds, and programmed cell death (Asad et al., 2019). These changes are associated with increases in intracellular reactive oxygen species (ROS) (Ramel et al., 2009). Insufficient light will accelerate leaf senescence and result in the excessive accumulation of ROS, oxidative damage to proteins, nucleic acids, and membrane lipids (Jbir-Koubaa et al., 2015), and decreases or losses of the activities of various enzymes (Mittler, 2002; Gill and Tuteja, 2010). As a consequence, the integrity of the cell membrane can be disrupted (Benlloch-Gonzalez et al., 2015), and the normal functions of chloroplast and mesophyll cells are eventually damaged, with decreased photosynthetic electron transport efficiency (Feng et al., 2021). Plants have a complete protective system of antioxidant enzymes that remove excessive ROS to protect the photosynthetic system and enhance adaptation to stress (Li et al., 2020).These enzymes include superoxide dismutase (SOD), and peroxidase (POD) (Dvorak et al., 2021). Thus, the antioxidant enzyme activity is an important indicator for evaluating whether the redox balance of plant cells is disrupted under adverse conditions (Gill et al., 2013).

The changes in the dependence of plant growth and development on light are regulated by plant hormones (Zhong et al., 2012). The changes in levels of plant hormones under shading are active responses by plants to adverse environments, and they provide the physiological basis that allows plants to make better use of assimilation products (Jiang et al., 2021). Gibberellin (GA) is necessary for the shade avoidance response in plants (Djakovic-Petrovic et al., 2007). In a previous study, we measured the changes in the contents of various hormones in two different light-sensitive inbred lines under shade treatment and in a control group without shade. According to the activities of the hormones, we found that the change in the gibberellin A_3_ (GA_3_) content was the most important. GA_3_ is a type of tetracyclic diterpene plant hormone that can regulate many plant growth and development processes (e.g., seed germination, stem elongation, pollen maturation and fruit development), and one of its important functions is regulating the flowering time (Vicente and Plasencia, 2011). Previous studies of *Arabidopsis* showed that endogenous GA was necessary for flowering under non-induced conditions, and the flowering time was generally delayed in a GA synthesis defect mutant and GA signal transduction mutant (Rood et al., 1989; Ni and Bradford, 1993; Magome et al., 2004). Exogenous GA application can also promote flowering in *Arabidopsis thaliana* (Ezura and Harberd, 1995; Sauret-Gueto et al., 2012; Bao et al., 2020). It should also be noted GA is an excellent antioxidant that can enhance the tolerance of various biological and abiotic stresses by plants (Yamaguchi et al., 2014; Khan et al., 2015). GA_3_ can also increase the number of cell divisions by activating the intermediate meristems to promote cell division (Mcatee et al., 2013). Guo et al. (2022) showed that spraying GA_3_ could improve photosynthesis and the antioxidant defenses to increase the yield in salted wood pea. The exogenous application of GA_3_ can delay the degradation of chlorophyll and protein, reduce the malondialdehyde (MDA) content, and delay plant senescence (Yu et al., 2009; Wang et al., 2015). Under low light stress, the abscisic acid (ABA) and zeatin (ZT) contents of soybean leaves decreased, whereas the indole acetic acid (IAA) and gibberellin (GA_3_) contents increased. Similar results were obtained in previous studies of maize leaves under low light conditions, thereby suggesting that the response of this hormone to low light is an active stress response that allows plants to adapt to low light environments. Therefore, it is of great theoretical and practical significance to study the regulatory effects of exogenous GA_3_ on different light-sensitive inbred maize lines under low light condition.

During the breeding of inbred maize lines over numerous years, we found and bred two inbred maize lines called SN98A and SN98B with extreme differences in their culms. In particular, the distinction, SN98A is called the “ear differentiation and sensitive to low light intensity inbred line”(ESL) and SN98B is called the “ear differentiation and insensitive to low light intensity near isogenic line” (EISL-NIL). Under low light stress condition, the hollowing rate in SN98A was 98% and that in SN98B was 0. Thus, in the present study, we used these weak light sensitive near-isogenic lines as experimental materials. By applying GA_3_ to leaves, the regulation effect of exogenous GA_3_ on empty stalk of maize under low light condition was analyzed. Through the analysis of photosynthetic response, antioxidant enzyme activity and other indexes, the purpose was to find out which physiological indexes of maize were affected by low light stress to induce maize stalk emptying. The regulatory effect of exogenous GA_3_ on maize hollows under low light conditions and its regulatory mechanism were discussed, so as to provide solutions for poor maize yield under bad weather conditions.

## Materials and methods

### Plant materials and experimental design

The maize varieties used in this study were SN98A and SN98B, which are inbred maize lines with extreme differences in the frequency of hollow culms. Under certain low light conditions, the hollow culm rate in SN98A is as high as 98%, whereas SN98B exhibits a normal ear setting. A field experiment was conducted at the South Experimental Base of Shenyang Agricultural University (41°48′N, 123°34′E) in July 2021. The normal light intensity from late July to early August in the Northern Test Field at Shenyang Agricultural University was usually between 1100− 1500 μmol m^–2^ s^–1^, with an average light intensity of about 1300 μmol m^–2^ s^–1^. Soil characteristics for temperate subhumid continental climate, the climate belongs to the temperate monsoon climate. A split block design was applied in the experiment. The concentration of GA_3_ was the main influence factor and the inbred line was the secondary influence factor. The length of the plot was 5 m, the row spacing was 0.6 m, and each plot had 15 rows. In the first three days of the tasseling period, 38% shading was applied with a black shade net, and different concentrations of GA_3_ were applied by spraying (Beijing Merida Technology Co., Ltd, China), 20mg L^-1^, 40mg L^-1^, and 60mg L^-1^ or water as the control. Samples were taken at five, 10, 15, and 20 days after shading, which were frozen in liquid nitrogen and then stored at -80°C. Each treatment was repeated three times.

### Phenotypic evaluations

The number of plants, number of bearing plants (with more than 20 grains), and the number of plants with empty stems under each treatment determined during the harvest period. The seed setting rate (%) and empty stalk rate (%) were calculated. Seed setting rate (%) = number of seeds/total number of plants *100%. Empty stalk rate (%) = number of empty culms/total number of plants *100%.

### Gas-exchange parameters

Net photosynthetic rate, stomatal conductance, transpiration rate and intercellular carbon dioxide concentration of panicle leaves under different treatments were measured by Li-6400 (US-COR) portable photosynthesometer at 5, 10, 15 and 20 days after shading (Zhou et al., 2022). The measuring time was 9:00-11:00 a.m. The measured environment was 400mol (CO_2_) mol^–1^ and 50% relative humidity. Ten replicates per process.

### Photosynthetic pigment contents

To determine the photosynthetic pigment contents (chlorophyll a (Chl a) and chlorophyll b (Chl b)), 0.1g of fresh leaves were crushed, soaked in 10 mL of acetone, and kept in the dark for 48 h. Chl was extracted and analyzed according to the methods reported by Lichtenthaler and Wellburn (1983). The absorbance values were then recorded at 645 and 663 nm by using a spectrophotometer (Multiskan GO, Thermo Fisher Science, USA), with acetone as a blank control. The following formulae were used to calculate the photosynthetic pigment contents:

Chl a [mg g^–1^ (FM)] = (12.7 × OD663–2.69 × OD645) ×V/M (1000×M)

Chl b [mg g^–1^ (FM)] = (22.9 × OD645–4.68 × OD663) × V/M (1000×M)

Chl (a+b) [mg g^–1^(FM)] = Chl a + Chl b

where OD645 and OD663 represent the absorbance values for Chl a/b at the corresponding wavelengths, V represents the total volume of the extract, and M represents the mass of the sample. Each treatment was repeated three times.

### Determination of chlorophyll fluorescence parameters

The panicle leaves were removed 20cm from the tip, placed in wet gauze, and stored for 30 min under certain humidity in the dark and away from light. The FluorCam (Czech PSI) chlorophyll fluorescence imaging system was used to determine the photochemical efficiency of photosystem II (PS II) (Fv/Fm). Chlorophyll fluorescence parameters were determined comprising the photochemical quenching coefficient (qP) and non-photochemical quenching coefficient (NPQ) and images were collected. Each treatment was repeated three times.

### Determination of H_2_O_2_ content and antioxidant enzyme activities

POD (extinction coefficient = 25.2 mm^–1^ cm^–1^) was determined at 470 nm in a 1.0 mL reaction mixture containing 100 mM potassium phosphate buffer (pH 6.0), 16 mM guaiacol, 5 μL 10% (v/v) H_2_O_2,_ and enzyme extract. The SOD activity was measured based on its capacity to inhibit blue light in the chemical reduction of nitrotetrazolium, which was monitored at 560 nm (Abedi and Pakniyat, 2010). Three biological replicates were tested for each sample.

### Determination of MDA and superoxide radical contents

The MDA content was determined by using the thiobarbituric acid method to evaluate the level of lipid peroxidation. Leaf tissue (0.5 g) was homogenized in 5.0 mL of 10% trichloroacetic acid and centrifuged at 4°C and 10,000×*g* for 10 min. The supernatant was assessed as described by Hodges et al. (1999). According to the method of Xia et al. (2009), with some modifications. The panicle leaves were sampled, cleaned with distilled water, and sucked dry. They were then placed in 50 mL 0.5 mg mL−1 NBT reaction solution (potassium phosphate buffer, pH 7.8) and incubated in darkness at 25°C for 2 h to detect ${\text{O}}_{2}^{–}$ . Three biological replicates were tested for each sample.

### Real-time fluorescence quantitative PCR detection of expressed of target genes

The *ZmActin* gene in maize was used as the internal reference gene and SYBR Green Real-time PCR Master Mix was used as the fluorescent dye. The samples were tested after shading for 15 days. The template comprised cDNA diluted 20 times and it was repeated three times. The total reaction system volume was 20 μL and the reaction conditions comprised: 95°CC for 30 s, and 45 cycles at 60°CC for 30 s and 72°CC for 30s (Xu et al., 2023). After PCR, the dissolution curve was analyzed. The primers used are shown in **Tables 1**, **2**.

**Table 1:** Primers used real-time fluorescence quantitative PCR.

Primer name	Primer (5’-3’)
*ZmActin*	F:GTTAAAGATTGCGCCACCT.R:GCCTGACGTACCATGTCGAAC
*APX*	F:CGCGCATTTCCAGATCTTTG.R:GATCGATGCGAGATCAGGGG
*Fe-SOD*	F:CGACTGTCCCTTCTCACAAA.R:ATCCGGTAAGGGACCTTCTT
*GR*	F:TTGGCAATGAACCTACCAAA.R:CAATTGCCTGCTCCTCAGTA

**Table 2:** Primers used real-time fluorescence quantitative PCR.

Primer name	Primer(5’-3’)
*DELLA1*	F:GCAAATCAAGCCATCCTC. R:AGCAAACGGCACTCTAACT
*DELLA2*	F:CAGGCGGTCCTCCTTCATTCC.R:GCTATCGCTTCTGGTTCCTCGTCGG
*DELLA3*	F:CAGCAACAGCAAGCCACA.R:CCACTTCTTCCACGCAATAC
*GID1C1*	F:CCCAATGGGAATGATCTCAA.R:ACAATTAGAACTCACAAAACCCTT
*GID1C2*	F:TCAACCCCACCCGAATCC.R:AGGTCGCCGTTGCATGTT
*GID1C3*	F:CAATTCACCCAATTCTAACC.R:AAATGCCTTCCAATACCAA
*GA20ox2*	F:CCCTCACCATTCTCCAACA.R:CCCGGACCACCTTATCTTC
*KAO1*	F:TTTGAAGGCAAGAAAGACG.R:TGTGATATGACCCGAAGAT
*KAO2*	F:ATGATTGACTTCTTGTGGTGCTT.R:TTAGACATCGCCGTAACCCCTT

### Statistical analysis

DPS (version 9.01) was used for multiple comparative analysis between treatments, the confidence level was 0.05, and one-way ANOVA was used. Data are expressed as mean standard deviation. Chart using Origin 2021 software.

## Results

### Seed setting rate and hollow stalk rate

**Figure 1:**
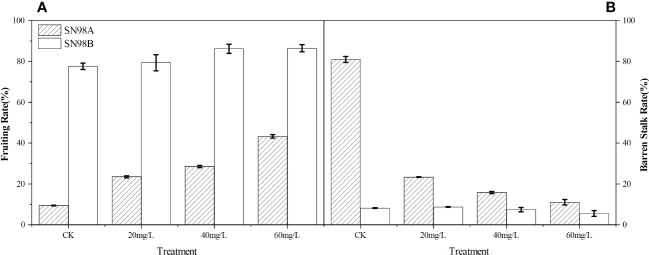
Effects of exogenous GA_3_ on seed setting rate and hollow stalk rate in different light-sensitive inbred maize lines under low light-stress. **(A)** Seed setting rates in different light-sensitive inbred maize lines sprayed with different concentrations of GA_3_ and water. **(B)** Hollow stalk rates in different light-sensitive inbred maize lines sprayed with different concentrations of GA_3_ and water. CK is the control water spraying treatment under shade. The numbers 20, 40, and 60 denote GA_3_ concentrations of 20 mg L^–1^, 40 mg L^–1^, and 60 mg L^-1^, respectively. SN98A is the shade intolerant line and SN98B is the shade tolerant line. Values are expressed as mean ± SD of three replicates. Lower-case letters indicate the mean difference of different treatments in the same period, which is statistically significant (P<0.05).

**Figure 1A** shows that after spraying GA_3_, the seed setting rate increased in the two inbred lines and the hollow stalk rate decreased. The seed setting rates were highest in the 60 mg L^–1^ GA_3_ treatment groups, where those in SN98A and SN98B were 23.56% and 14.68% higher than that in the control group sprayed with water, respectively (Fig 1A).Treatment with GA_3_ at 60 mg L^–1^ obtained the lowest hollowing rates, where the rates were 14.25% and 12.34% lower in SN98A and SN98B than the controls, respectively (**Figure 1B**).

### Photosynthetic parameters

**Figure 2:**
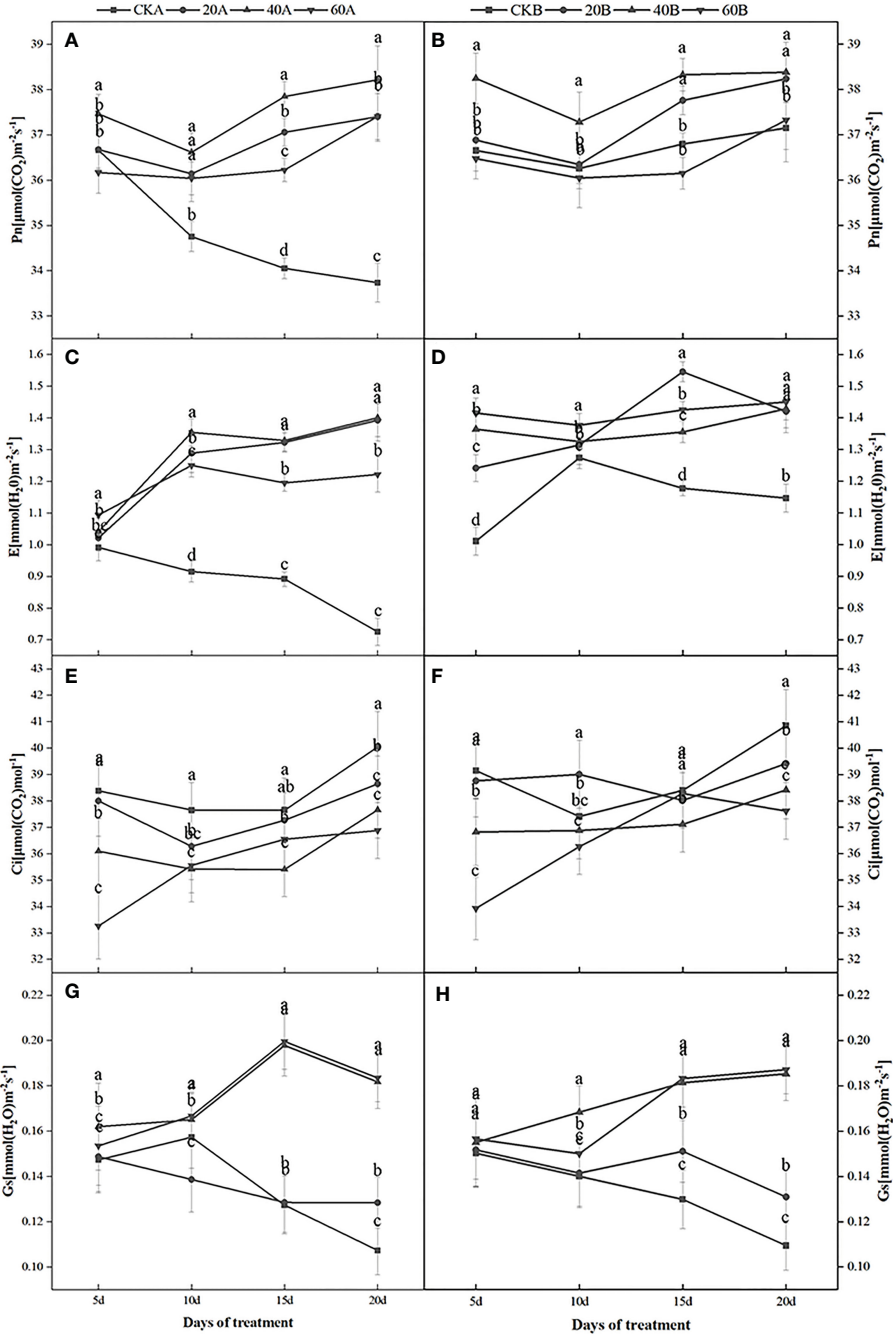
Effects of GA_3_ on net photosynthetic rate (Pn), transpiration rate (Tr), intercellular CO_2_ concentration, and stomatal conductance (Gs) in different light-sensitive inbred maize lines under low light stress. **(A, B)** show the Pn values; **(C, D)** show the Tr values; **(E, F)** show the intercellular carbon dioxide concentrations; and **(G, H)** show the Gs values. In the figure panels, 20A, 40A, and 60A denote SN98A sprayed with 20 mg L^–1^,40 mg L^–1^, and 60 mg L^–1^ GA_3_, respectively, and 20B, 40B, and 60B denote SN98B sprayed with 20 mg L^–1^,40 mg L^–1^,and 60 mg L^–1^ GA_3_.CKA denotes the SN98A control group sprayed with water and CKB denotes the SN98B control group sprayed with water. Values are expressed as mean ± SD of three replicates. Lower-case letters indicate the mean difference of different treatments in the same period, which is statistically significant (P<0.05).

**Figure 2** shows that in the control group under low light weak light conditions, Pn continued to decrease in SN98A, whereas Pn in SN98B tended to decrease initially before then increasing. Among the two inbred lines, Pn was always higher in SN98B than SN98A. After GA_3_ treatment, Pn and the transpiration rate (Tr) were significantly higher in SN98A compared with the control. After shading for 20 days, the mean Pn and Tr values in the three treatment groups were 10.88% and 68.43% higher than those in the control, respectively. Thus, GA_3_ treatment had a positive regulatory effect on Pn in the low light-sensitive inbred line SN98A. Treatment with 40 mg L^–1^ GA_3_ had the greatest effect but the difference between the treatments was not significant (**Figure 2**). After GA_3_ treatment, the stomatal conductance (Gs) was generally higher in the two inbred lines than the control, and the external application of 60 mg L^–1^ GA_3_ had the greatest effect. The intercellular CO_2_ concentrations in the two inbred lines were also lowest at this concentration. Thus, the external application of GA_3_ under low light conditions increased Gs for the maize leaves and enhanced the photosynthetic activity of the mesophyll cells according to the Pn results. Pn increased under low light conditions in SN98A.

### Chlorophyll fluorescence parameters

**Figure 3:**
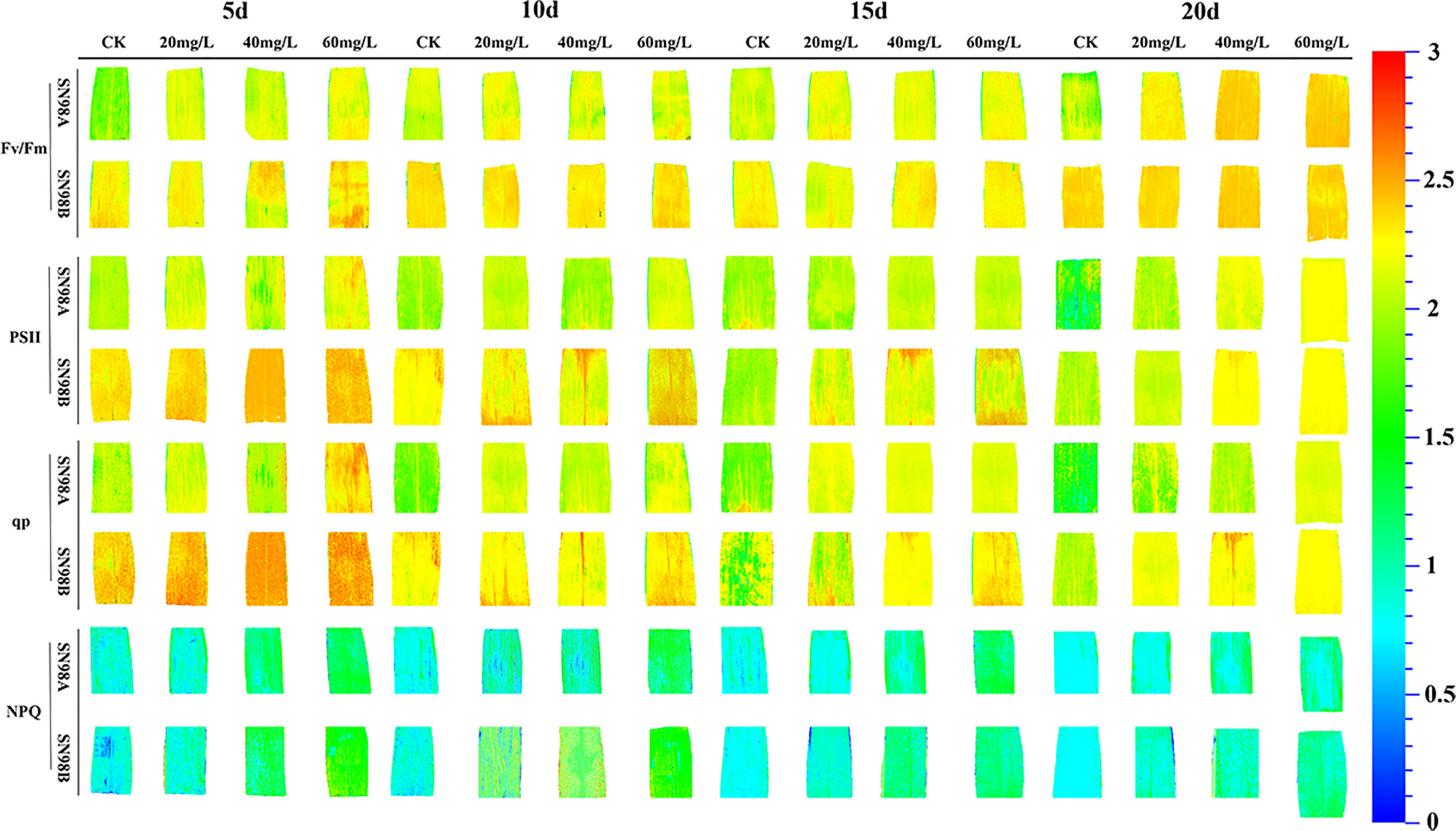
Effects of GA_3_ application on chlorophyll fluorescence according to *in situ* imaging of inbred maize lines with differences in light sensitivity under low light stress conditions. In the figure panels, 5d, 10d, 15d, and 20d denote the number of days under shading treatment; 20, 40, and 60mg L^–1^ denote the GA_3_ concentrations applied; and CK denotes the water control treatment.

After shading treatment, Fv/Fm, the effective quantum yield of PSII photochemistry (Φ_PSII_), and qP were significantly lower in the SN98A control group than SN98B, whereas the NPQ values were significantly higher compared with SN98B. Compared with the control, GA_3_ significantly improved the PSII photosynthetic characteristics of maize leaves, where treatment with 60 mg L^–1^ GA_3_ had the greatest effect, and SN98A had the highest Fv/Fm, Φ_PSII_ and qP values. On day 20 under 60 mg L^–1^ GA_3_ treatment, the Fv/Fm, Φ_PSII_, and qP values in SN98A were 6.9%, 16.39%, and 14.75% higher, respectively, compared with those in the control, and the NPQ value was 22.89% lower compared with the control (**Figure 3**). The effect of GA_3_ treatment on SN98B was not significant, but the photosynthetic activity of PSII was higher than that in SN98A, thereby indicating that GA_3_ was involved in the shading reaction by maize leaves and it had a positive role in maintaining the photosynthetic efficiency of maize leaves under low light stress.

### Chlorophyll contents

**Figure 4:**
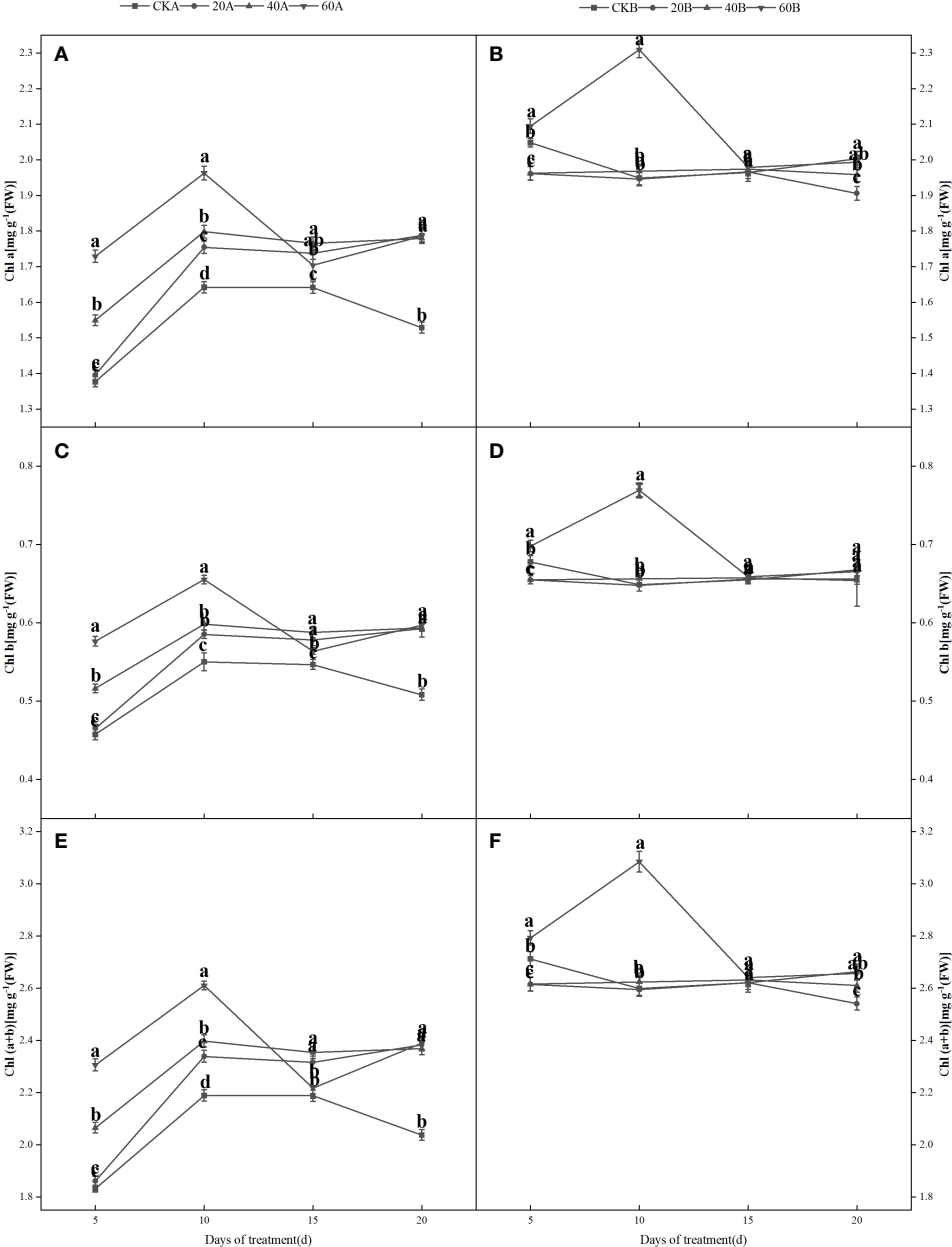
Effects of GA_3_ application on the Chl a **(A, B)**, Chl b **(C, D)**, and Chl a+b **(E, F)** contents of different light-sensitive inbred maize lines under low light stress. Values are expressed as mean ± SD of three replicates. Lower-case letters indicate the mean difference of different treatments in the same period, which is statistically significant (P<0.05).

**Figure 4** shows that under shading treatment, the Chl a, Chl b and Chl (a+b) contents in the SN98A control group tended to increase initially and then decrease, whereas the Chl content in SN98B did not significantly and it was always higher than that in SN98A. Compared with the control, GA_3_ had a significant positive regulatory effect on the photosynthetic pigment contents of the low light-sensitive inbred line SN98A, and the Chl a, Chl b and Chl (a+b) contents increased under all three GA_3_ treatments. The effect of treatment with 60 mg L^–1^ GA_3_ was most obvious, and the photosynthetic pigment contents increased on days 5, 10, 15, and 20 after treatment, where the Chl (a+b) contents increased by 25.7%, 19.6%, 2.9% and 17.3%, respectively. The photosynthetic pigment content of SN98B was the same as that of the control and it was always higher than that of SN98A, where the content was significantly higher in SN98B on day 10 after treatment with 60 mg L^–1^ GA_3_.

### ROS contents and membrane lipid peroxidation

**Figure 5:**
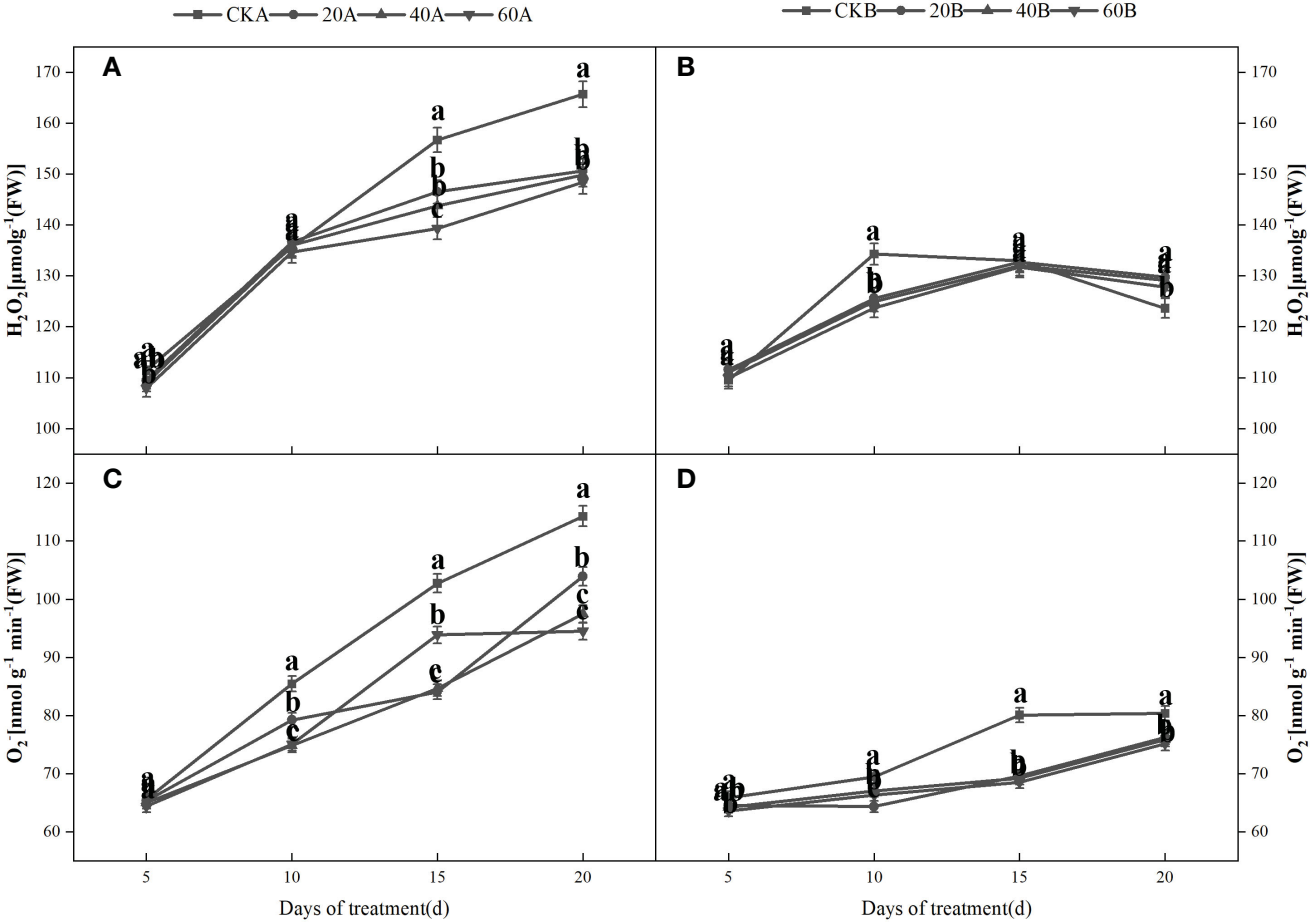
Effects of GA_3_ on H_2_O_2_ contents and ${\text{O}}_{2}^{–}$ production rate in light-sensitive inbred maize lines after different periods under low light stress. In the figure panels, 20A, 40A, and 60A denote SN98A sprayed with 20 mg L^–1^, 40 mg L^–1^, and 60 mg L^–1^ GA_3_, respectively, and 20B, 40B, and 60B denote SN98B sprayed with 20 mg L^–1^, 40 mg L^–1^, and 60 mg L^–1^ GA_3_. CKA denotes the SN98A control group sprayed with water and CKB denotes the SN98B control group sprayed with water. In the figure, **(A**, **C)** represent the changes of H_2_O_2_ and ${\text{O}}_{2}^{–}$ after SN98A is treated with GA_3_. **(B**, **D)** are the changes of H_2_O_2_ and ${\text{O}}_{2}^{–}$ after SN98B is treated with GA_3_. Values are expressed as mean ± SD of three replicates. Lower-case letters indicate the mean difference of different treatments in the same period, which is statistically significant (P<0.05).

**Figure 6:**
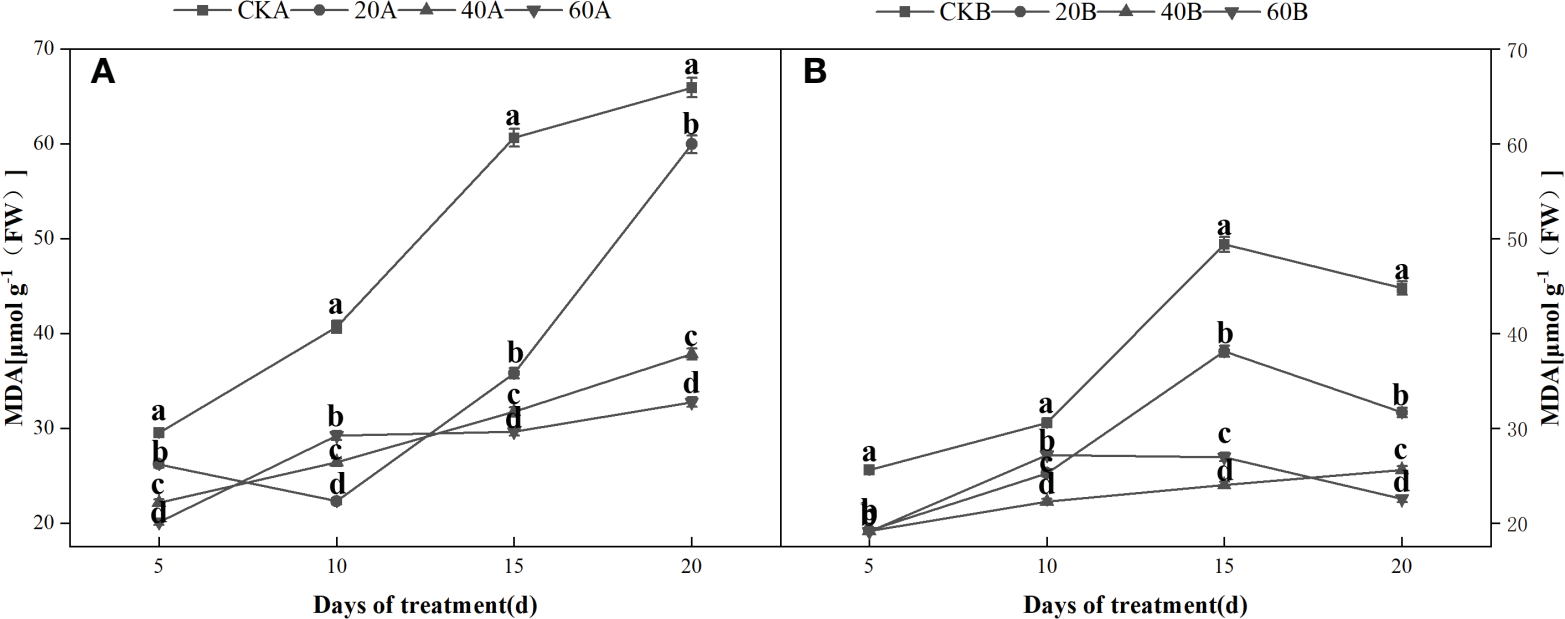
Effects of GA_3_ on MDA content of light-sensitive maize inbred lines at different periods under low light stress. In the figure panels, 20A, 40A, and 60A denote SN98A sprayed with 20 mg L^–1^, 40 mg L^–1^, and 60 mg L^–1^ GA_3_, respectively, and 20B, 40B, and 60B denote SN98B sprayed with 20 mg L^–1^, 40 mg L^–1^, and 60 mg L^–1^ GA_3_. CKA denotes the SN98A control group sprayed with water and CKB denotes the SN98B control group sprayed with water. In the figure, **(A)** is the change of MDA in SN98A after GA_3_ treatment, and **(B)** is the change of MDA in SN98B after GA_3_ treatment. Values are expressed as mean ± SD of three replicates. Lower-case letters indicate the mean difference of different treatments in the same period, which is statistically significant (P<0.05).

Under shading, the H_2_O_2_ and ${\text{O}}_{2}^{–}$ contents increased initially and then decreased in the SN98B control group, whereas the H_2_O_2_ and ${\text{O}}_{2}^{–}$ contents continued to increase in the hollowing line SN98A and the contents were always higher than those in SN98B (**Figure 5**). After GA_3_ treatment, the H_2_O_2_ and ${\text{O}}_{2}^{–}$ contents of the two inbred lines were lower compared with those in the control group. After shading for 15days, treatment with 60 mg L^–1^GA_3_ significantly reduced the ${\text{O}}_{2}^{–}$ production rate and H_2_O_2_ content of SN98A by 17.3% and 10.4%, respectively. In the control and treatment groups, the H_2_O_2_ and ${\text{O}}_{2}^{–}$ contents were always higher in SN98A than SN98B, and the ROS contents were always lower in SN98B. The changes in the ROS contents (H_2_O_2_ and ${\text{O}}_{2}^{–}$ ) were basically the same. GA_3_ treatment significantly reduced the MDA contents of the two inbred lines under low light. The MDA contents were higher in SN98A than SN98B in both the control and treatment groups (**Figure 6**). The effects of short-term shading were similar under the three treatments, but the effect of treatment with 20 mg L^–1^ GA_3_ decreased as the shading period continued. In conclusion, the application of GA_3_ could have reduced the peroxidation of membrane lipids caused by the accumulation of ROS in maize leaves to delay leaf senescence and enhance the tolerance of shading in maize. Treatments with 40 mg L^–1^ and 60 mg L^–1^ GA_3_ were most effective.

### Antioxidant enzyme activities

**Figure 7:**
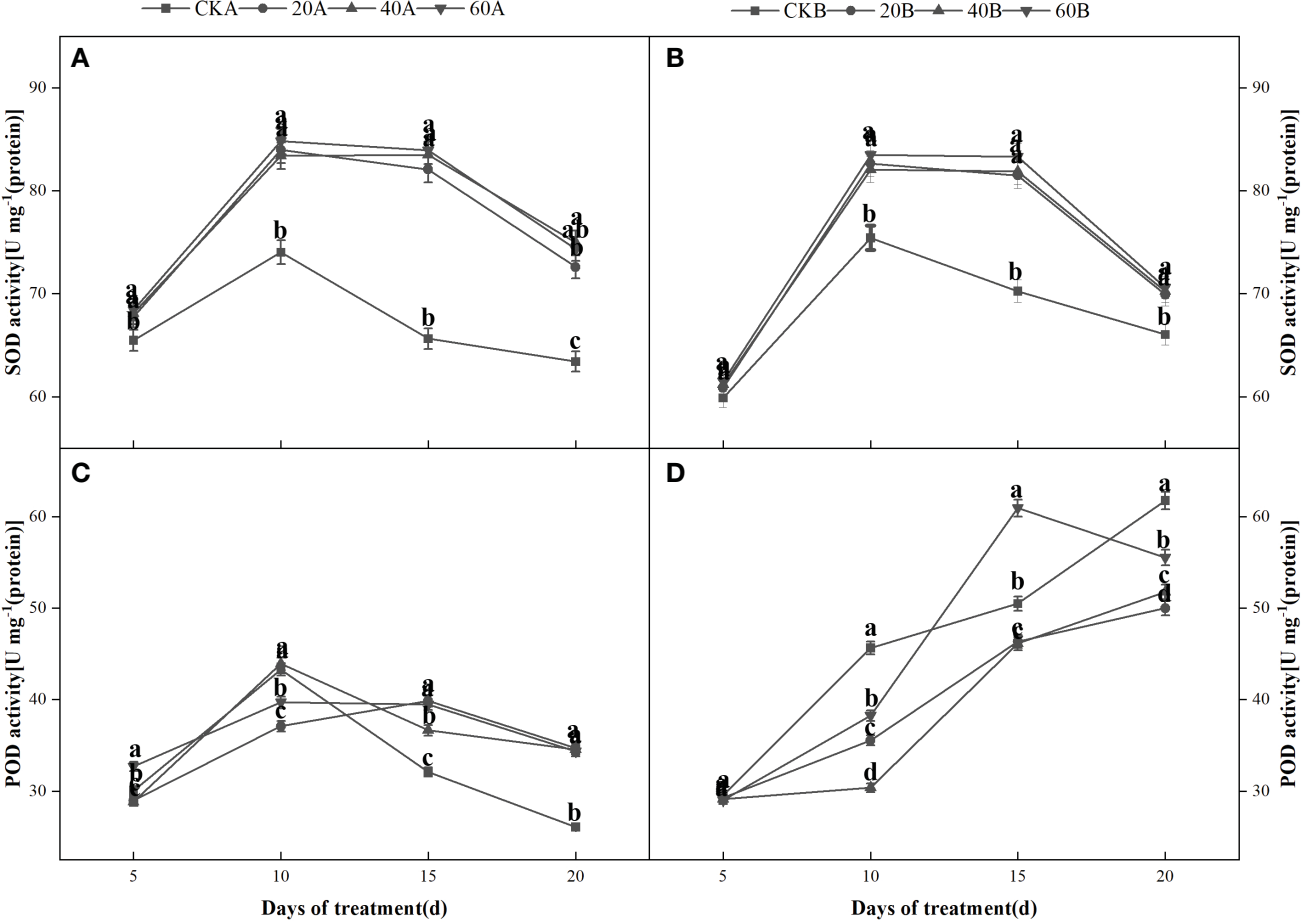
Effects of GA_3_ on antioxidant enzymes contents in different light-sensitive inbred maize lines after different periods under low light stress. In the figure panels, 20A, 40A, and 60A denote SN98A sprayed with 20 mg L^–1^, 40 mg L^–1^, and 60 mg L^–1^ GA_3_, respectively, and 20B, 40B, and 60B denote SN98B sprayed with 20 mg L^–1^, 40 mg L^–1^, and 60 mg L^–1^ GA_3_. CKA denotes the SN98A control group sprayed with water and CKB denotes the SN98B control group sprayed with water. In the figure, **(A, C)** represent the changes of H_2_O_2_ and ${\text{O}}_{2}^{–}$ after SN98A is treated with GA_3_. **(B, D)** are the changes of H_2_O_2_ and ${\text{O}}_{2}^{–}$ after SN98B is treated with GA_3_. Values are expressed as mean ± SD of three replicates. Lower-case letters indicate the mean difference of different treatments in the same period, which is statistically significant (P<0.05).

After shading and treatment with different concentrations of GA_3_, the SOD and POD activities increased initially and then decreased in the SN98A control group, where the activities were highest after shading for 10 days. The POD activities continued to increase in SN98B and did not peak until 20 days. During short-term shading the antioxidant system was activated to remove ROS and maintain crop growth. Crops can avoid damage caused by short-term adverse conditions by activating their stress response mechanisms, but they cannot prevent damage under long-term adverse conditions. After treatment with GA_3_ at different concentrations, the antioxidant enzyme activities were significantly higher in SN98A than the control (**Figure 7**), where treatment with 60 mg L^–1^ GA_3_ had the most significant effect. After shading for 20 days, the SOD and POD enzyme activities in SN98A were 17% and 31.7% higher compared with the control, respectively, and the difference was significant (**Figures 7A, C**).

### Antioxidant-related genes

**Figure 8:**
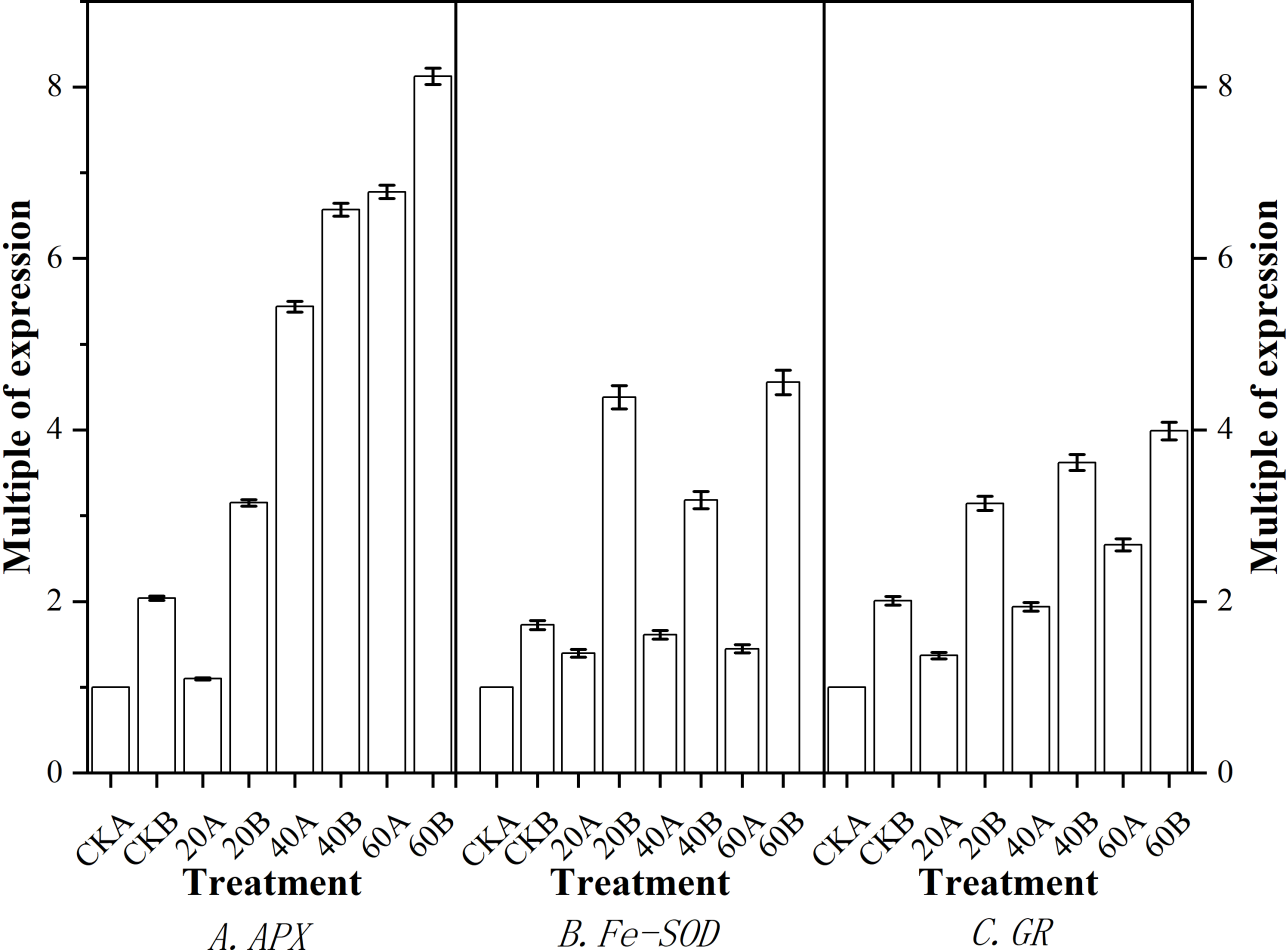
Effects of GA_3_ application under low light stress on the expression levels of antioxidant-related genes in different light-sensitive inbred maize lines after shading for 15 days. **(A)** *APX*; **(B)** *Fe-SOD*; **(C)** *GR*. Values are expressed as mean ± SD of three replicates. Lower-case letters indicate the mean difference of different treatments in the same period, which is statistically significant (P<0.05).

Real-time fluorescence quantitative PCR analysis was performed to quantify the expression levels of three genes related to antioxidant stress, and the results are shown in **Figure 8**. Compared with the control, after GA_3_ treatment, the expression levels of *APX* (**Figure 8A**) and *GR* (**Figure 8C**) were significantly higher in SN98A under treatment with different GA_3_ concentrations, where the expression levels were highest under treatment with 60 mg L^–1^ GA_3_, i.e., 6.73 and 2.75 times than those in the control, respectively. The expression levels of *APX* and *GR* under treatment with 40 mg L^–1^ GA_3_ were 5.41 times and 1.93 times those in the control, respectively. The expression of *Fe-SOD* (**Figure 8B**) did not increase significantly in SN98A, where the expression level was highest under treatment with 40 mg L^–1^ GA_3_ i.e., 1.62 times that in the control. All three genes responded positively to exogenous GA_3_ in SN98B, whereas only *APX* and *GR* responded positively in SN98A.

### GA-related gene

**Figure 9:**
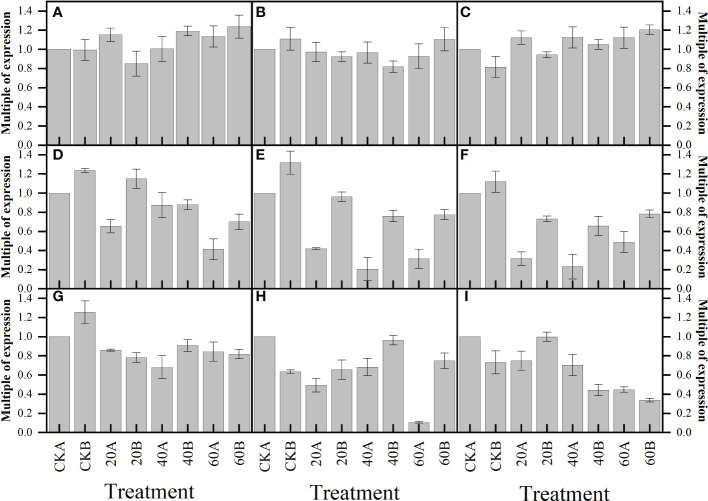
Effects of GA_3_ application under low light stress on GA-related gene expression levels of different photosensitive inbred lines after shade for 15 days. **(A)** DELLA1; **(B)** DELLA2; **(C)** DELLA3; **(D)** GID1C1; **(E)** GID1C2; **(F)** GID1C3; **(G)** GA20ox2; **(H)** KAO1; **(I)**KAO2. Values are expressed as mean ± SD of three replicates.

In this experiment, the relative expressions of gibberellin receptors *GID1C1*, *GID1C2* and *GID1C3* decreased after exogenous GA_3_ treatment, and the relative expressions of *GA20ox*, *KAO1* and *KA02* genes related to gibberellin synthesis and degradation decreased in plants (**Figure 9**). The expression levels of signal transduction related genes *DELLA1*, *DELLA2* and *DELLA3* were not significantly changed. These results indicated that exogenous GA_3_ could inhibit the synthesis of endogenous gibberellin in maize.

## Discussion

Northeast China is the most important maize-producing area in China, where the maize output in this region account for more than 30% of the national maize output. In this region, light, heat, and water resources are generally abundant in the growing period. However due to global climate change, extreme weather events have become more frequent during the maize growing season, The frequency of overcast weather with high rain and low radiation during the withering and silking stage has recently increased each year (Yang et al., 2020), thereby adversely affecting the stability of the maize yield in Northeast China. Light is essential for photosynthesis by plants and the basis for plant growth and development (Jiang et al., 2021). Maize is a light-loving crop with no obvious light saturation point and it is very sensitive to changes in the light intensity. A lack of light during tasseling will inhibit the normal physiological activities, decrease photosynthesis in the leaves, and cause cell metabolism disorders and REDOX homeostasis damage, thereby leading to problems such as ear bald tip, yield reductions, and even hollow stalks. Low light stress can accelerate the excessive accumulation of ROS in maize leaves and cause the peroxidation of membrane lipids. Colebrook et al. (2014) indicated that hormones can effectively help plants to cope with abiotic stress, where stress responses are regulated mainly by activating specific hormones *via* signal transduction and crosstalk in different developmental environments (Verma et al., 2016). Therefore, how to increase the yield and reduce the empty stalk rate under the condition of insufficient light has become an important problem.

GA_3_ is a plant growth regulator and it is involved in the response to many abiotic stresses in plants, with positive regulatory effects on plant growth and development (Bao et al., 2020). Exogenous GA_3_ application can reduce the effects of nickel stress (Wiszniewska et al., 2018) and increase the plant cell length independent of biosynthesis (Rizza et al., 2017). In addition, GA and photoperiod pathways have been shown to synergistically regulate crop flowering under long sunshine conditions (Fukuda et al., 2012; Wang et al., 2016). In the present study, GA_3_ treatment reduced the hollow stem rate in maize by regulating the flowering interval between male and female ears, where treatment with 60 mg L^–1^ GA_3_ had the greatest effect, followed by treatment with 40 mg L^–1^ GA_3_ and 20 mg L^–1^GA_3_. Under shading treatment, the H_2_O_2_ and ${\text{O}}_{2}^{–}$ contents continued to increase in the SN98A control group, and the contents were always higher than those in SN98B. After GA_3_ spraying treatment, the ${\text{O}}_{2}^{–}$ production rate and H_2_O_2_ contents still increased in SN98A and SN98B, but the increases were significantly smaller than those in the control group, and the ${\text{O}}_{2}^{–}$ production rate and H_2_O_2_ contents were always lower in SN98B than SN98A due to the photoprotective capacity of the shade-tolerant inbred line SN98B. Low light stress can induce oxidative stress in maize, but GA_3_ treatment can enhance the ability of maize to resist low light stress and reduce the oxidative pressure caused by insufficient light. Numerous studies have shown that membrane lipid peroxidation leads to the accumulation of MDA. Low light stress can disrupt the dynamic equilibrium between ROS production and scavenging in plants, thereby resulting in the accumulation of ROS and increased membrane permeability. As a consequence, membrane lipid peroxidation lead to the accumulation of MDA and exacerbates maize aging (Wang et al., 2021). In this study, GA_3_ treatment significantly reduced the MDA contents of the two inbred lines under low light. The MDA contents were higher in SN98A than SN98B in both the control and treatment groups. The antioxidant enzyme system in plants protects against the toxic effects of ROS. SOD and POD are important antioxidant enzymes in the plant defense mechanism. GA_3_ may have reduced the ROS contents in maize by enhancing the activities of POD and SOD in the light-sensitive inbred line SN98A, as also suggested by Ali et al. (2021). *APX*, *Fe-SOD* and *GR* are important genes related to the activities of antioxidant enzymes (Lee et al., 2007). Studies have shown that the overexpression of *APX*, *Fe-SOD*, and *GR* significantly improved the tolerance of abiotic stresses by transgenic tall fescue plants. In the present study, we found that exogenous GA_3_ treatment significantly upregulated the expression levels of *APX* and *GR* in SN98A and SN98B compared with the control group, thereby indicating that exogenous GA_3_ could enhance the activities of antioxidant enzymes to resist external abiotic stress by increasing the expression of antioxidant-related genes. Therefore, exogenous GA_3_ may improve the tolerance of stress in plants by regulating antioxidant metabolism and reducing the lipid peroxidation of cell membranes (Gilroy and Jones, 1992; Jiang and Huang, 2001) to decrease the damage due to low light stress in plants. According to the expression level of GA-related genes, the gene pathway of gibberellin signaling is GA-GID1-DELLA signaling pathway. When gibberellin is at a high level, GID1 can sense the GA signal and combine with it to form GA-GID1. Then it binds to DELLA protein to form GID1-GA-DELLA complex trimer (Zentella et al., 2007), so that ubiquitin ligase SCF in F-Box protein can bind to DELLA protein GRAS region (Fleet and Sun, 2005). The rapid degradation of DELLA protein through ubiquitin proteomic channels resulted in the release of its repression and normal gibberellin response in plants. Therefore, the reduction of gibberellin receptor GID1 may also affect the degradation of DELLA protein at the protein level. After exogenous GA_3_ was applied, the expression of *GA20ox2*, a gene related to gibberellin synthesis, decreased (FIG 9G). *GA20ox2* plays an important role in the synthesis of gibberellin in higher plants, and the decreased expression of *GA20ox* gene may decrease the production of endogenous gibberellin. In CK group, the expression level of gibberellin-synthesis-related genes in SN98A was lower than that in SN98B, and CKA of degradation-related genes was higher than that in CKB. Therefore, low light stress inhibited the synthesis of gibberellin by decreasing the expression of genes related to gibberellin synthesis and enhancing the expression of genes related to degradation, resulting in empty culms.

The responses of photosynthetic organs to low light stress and the associated mechanisms are important for understanding the adaptation of crops to different light environments. Thus, many studies have investigated the self-regulation mechanism and light energy conversion process in maize under low light conditions (Zivcak et al., 2014; Hazrati et al., 2016; Yamori, 2016). Chloroplasts are the site of photosynthesis in plants. Weak light stress can significantly damage the anatomical structure of plants to directly affect normal photosynthetic electron transport and the photosynthetic rate (P_n_) (Du et al., 2011; Feng et al., 2021). Under low light, the oxidative pressure is intensified in maize leaves and the accumulation of large amounts of ROS leads to changes in the spatial configurations of various enzymes in chloroplasts, thereby adversely affecting their function, decreasing the chlorophyll content, and inhibiting photosynthesis. In the present study, under shading treatment, the Chl a, Chl b and chlorophyll (a+b) contents, tended to increase initially and then decrease in SN98A, whereas the Chl contents did not change greatly in SN98B and they were always higher than those in SN98A. After GA_3_ treatment, the photosynthetic pigment contents increased significantly in SN98, and the Pn, Tr, and Gs values were also significantly higher compared with those in the control. PSII is one of the primary sites in photosynthetic organs damaged by stress, and it plays important roles in the light energy conversion and electron transport processes. (Zhang et al., 2020). The photoreaction requires coordination of the photosynthetic system in order to complete the normal linear electron transfer and provide the homogeneity for the fixation and reduction of CO_2_ in the dark reaction. (Qian et al., 2017). In the present study, Pn, qP, and Φ_PSII_ continued to decrease during shading in SN98A, whereas they tended to increase initially and then decrease in SN98B, but the values were always higher than those in SN98A, thereby indicating that the normal photosynthetic physiological process was maintained in SN98B. GA_3_ treatment could have increased the stomatal conductivity of the maize leaves, enhanced the photosynthetic activity of mesophyll cells, and improved the photosynthetic capacity of maize leaves (Verma et al., 2016), where 60 mg L^–1^ GA_3_ treatment had the greatest effect, and thus GA_3_ may be involved in the shade protection response process in maize leaves. GA_3_ had a positive effect on the photosynthetic efficiency of maize leaves under low light stress.

Plant photosynthesis is an extremely complex physiological process and it is negatively affected by both biological and abiotic stresses. The main limiting factors for photosynthesis are light and carbon dioxide (Smith, 1938). Under low light stress, photosynthesis is inhibited in crops and yields are reduced (Liu et al., 2020). In the present study, under low light stress, Pn, Tr, and Gs all decreased with time during shading, in the SN98A control group and the intercellular carbon dioxide concentration increased. Applying GA_3_ improved Pn, Tr, and Gs in SN98A and reduced the intercellular carbon dioxide concentration, where the effect of 60 mg L^–1^ GA_3_ was most significant. Low light stress also reduced Pn, Tr, and Gs in the leaves in the SN98B control group, and increased the intercellular carbon dioxide concentration, but the normal physiological activities were generally maintained.

## Conclusion

**Figure 10:**
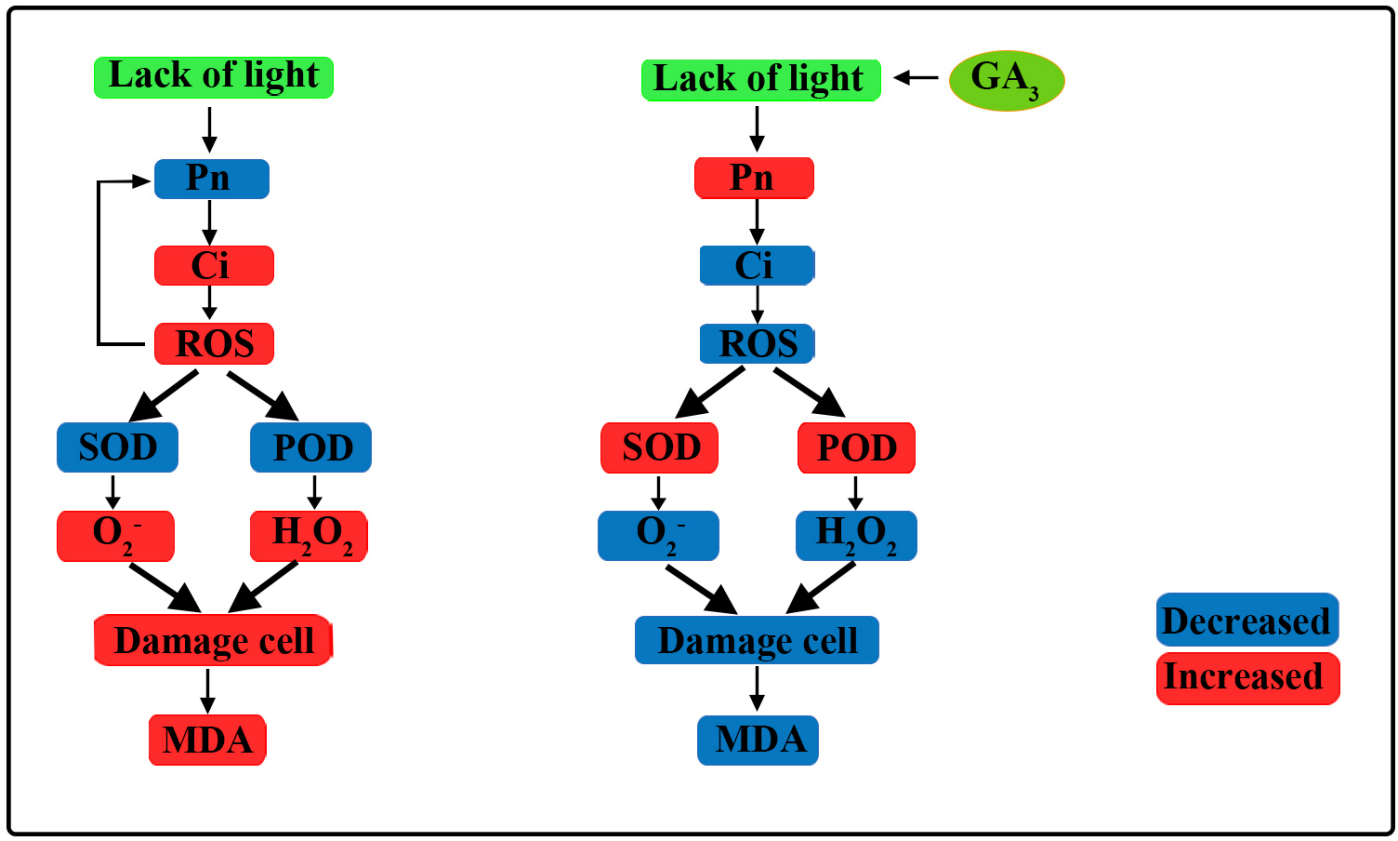
Overview of effects of exogenous GA_3_ on plant photosynthetic physiology and reactive oxygen species (ROS) scavenging pathways. Under low light, the net photosynthetic rate (Pn) decreased, the intercellular carbon dioxide concentration increased, and the ROS content increased, which further affected Pn. Under low light, the activities of antioxidant enzymes decreased and the ROS scavenging rate decreased, thereby leading to cell damage and MDA was produced increase the degree of membrane lipid peroxidation. After the application of GA_3_, Pn improved, the activities of antioxidant enzymes increased, ROS scavenging was enhanced, cell damage was reduced, and the MDA content decreased.

In the present study, maize inbred lines SN98A and SN98B with differences in their light sensitivity were subjected to shading in the maize tasseling stage (**Figure 10**). We found that low light treatment reduced the photosynthetic capacity of the maize leaves and inhibited the transport of photosynthetic products to other organs, thereby resulting in plant growth inhibition and leaf senescence. The adverse effects of shading were significantly greater in the weak light-sensitive inbred line SN98A than SN98B, mainly because SN98A had a significantly higher hollow stem rate than SN98B after shading. GA is an excellent antioxidant that can improve the tolerance of various biological and abiotic stresses in plants. These findings help us to understand the physiological mechanisms in maize inbred lines with differences in photosensitivity that mediate the response to low light stress under treatment with exogenous GA. The photosynthetic performance parameters of plants, i.e., Pn, Tr, Gs, photosynthetic pigment contents, (Fv/Fm), qP, and Φ_PSII_, improved after applying GA_3_ (20 mg L^–1^, 40 mg L^–1^, and 60 mg L^–1^) to the leaves, including 60 mg L^-1^ GA_3_ works best. Thus, the foliar application of GA_3_ to SN98A and SN98B will be beneficial for their growth and development under lower light conditions.

## Data availability statement

The original contributions presented in the study are included in the article/**Supplementary Material**. Further inquiries can be directed to the corresponding author.

## Author contributions

JF: investigation, data curation, validation, and writing—original draft; LL: review and editing; SW: data curation, methodology, formal analysis, and software; NY: formal analysis and editing; HS: investigation and formal analysis; ZS: resources and funding acquisition; FL: writing—review and editing; XZ: conceptualization, writing—review, editing and funding acquisition. All authors contributed to the article and approved the submitted version.

## References

[B1] Abedi T. Pakniyat H. (2010). Antioxidant enzyme changes in response to drought stress in ten cultivars of oilseed rape (Brassica napus l.). Czech J. Genet. Plant Breed. 46 (1), 27–34. doi: 10.17221/67/2009-CJGPB

[B2] Ali M. Kamran M. Abbasi G. H. Saleem M. H. Ahmad S. Parveen A.. (2021). Melatonin-induced salinity tolerance by ameliorating osmotic and oxidative stress in the seedlings of two tomato (*Solanum lycopersicum* l.) cultivars. J. Plant Growth Regul. 40, 2236–2248. doi: 10.1007/s00344-020-10273-3

[B3] Asad M. Zakari S. A. Zhao Q. Zhou L. Ye Y. Cheng F. (2019). Abiotic stresses intervene with ABA signaling to induce destructive metabolic pathways leading to death: Premature leaf senescence in plants. Int. J. Mol. Sci. 20 (2), 256. doi: 10.3390/ijms20020256 30634648 PMC6359161

[B4] Bao S. J. Hua C. M. Shen L. S. Yu H. (2020). New insights into gibberellin signaling in regulating flowering in arabidopsis. J. Integr. Plant Biol. 62, 118–131. doi: 10.1111/jipb.12892 31785071

[B5] Benlloch-Gonzalez M. Quintero J. M. Garcia-Mateo M. J. Fourniera J. M. Benllocha M. (2015). Effect of water stress and subsequent re-watering on k+ and water flows in sunflower roots. A possible mechanism to tolerate water stress. Environ. Exp. Bot. 118, 78–84. doi: 10.1016/j.envexpbot.2015.06.008

[B6] Colebrook E. H. Thomas S. G. Phillips A. L. Hedden P. (2014). The role of gibberellin signalling in plant responses to abiotic stress. J. Exp. Biol. 217, 67–75. doi: 10.1242/jeb.089938 24353205

[B7] Djakovic-Petrovic T. De Wit M. Voesenek L. Pierik R. (2007). DELLA protein function in growth responses to canopy signals. Plant J. Cell Mol. Biol. 51, 117–126. doi: 10.1111/j.1365-313X.2007.03122.x 17488236

[B8] Du C. Li C. Liu T. Zhao Y. (2011). Response of anatomical structure and photosynthetic characteristics to low light stress in leaves of different maize genotypes. Acta Ecologica Sinica. 21, 6633–6640.

[B9] Dvorak P. Krasylenko Y. Zeiner A. Samaj J. Takac T. (2021). Signaling toward reactive oxygen species-scavenging enzymes in plants. Front. Plant Sci. 11. doi: 10.3389/fpls.2020.618835 PMC7882706 33597960

[B10] Ezura H. Harberd N. P. (1995). Endogenous gibberellin levels influence *in-vitro* shoot regeneration in arabidopsis thaliana (L.) heynh. Planta 197, 301–305. doi: 10.1007/BF00202651 8547816

[B11] Feng Y. Cui X. Shan H. Shi Z. S. Li F. H. Wang H. W.. (2021). Effects of solar radiation on photosynthetic physiology of barren stalk differentiation in maize. Plant Sci. 312. doi: 10.1016/j.plantsci.2021.111046 34620444

[B12] Ferrante A. Mariani L. (2018). Agronomic management for enhancing plant tolerance to abiotic stresses: High and low values of temperature, light intensity, and relative humidity. Horticulturae 4. doi: 10.3390/horticulturae4030021

[B13] Fleet C. M. Sun T.-P. (2005). A DELLAcate balance: the role of gibberellin in plant morphogenesis. Curr. Opin. Plant Biol. 8, 77–85. doi: 10.1016/j.pbi.2004.11.015 15653404

[B14] Fukuda N. Yoshida T. Olsen J. E. Senaha C. Jikumaru Y. Yuji K. (2012). “Short main shoot length and inhibition of floral bud development under red light can be recovered by application of gibberellin and cytokinin,” in 7th international symposium on light in horticultural systems. Acta Hortic. 956 (956), 215–222. doi: 10.17660/ActaHortic.2012.956.23

[B15] Gill S. S. Anjum N. A. Hasanuzzaman M. Gill R. Trivedi D. K. Ahmad I.. (2013). Glutathione and glutathione reductase: A boon in disguise for plant abiotic stress defense operations. Plant Physiol. Biochem. 70, 204–212. doi: 10.1016/j.plaphy.2013.05.032 23792825

[B16] Gill S. S. Tuteja N. (2010). Reactive oxygen species and antioxidant machinery in abiotic stress tolerance in crop plants. Plant Physiol. Biochem. 48, 909–930. doi: 10.1016/j.plaphy.2010.08.016 20870416

[B17] Gilroy S. Jones R. L. (1992). Gibberellic acid and abscisic acid coordinately regulate cytoplasmic calcium and secretory activity in barley aleurone protoplasts. Proc. Natl. Acad. Sci. United States America 89, 3591–3595. doi: 10.1073/pnas.89.8.3591 PMC48914 1533046

[B18] Guo X. Q. Wu Q. D. Zhu G. L. Ibrahim M. E. H. Zhou G. S. (2022). Gibberellin increased yield of sesbania pea grown under saline soils by improving antioxidant enzyme activities and photosynthesis. Agronomy-Basel 12. doi: 10.3390/agronomy12081855

[B19] Hazrati S. Tahmasebi-Sarvestani Z. Modarres-Sanavy S. Mokhtassi-Bidgoli A. Nicola S. (2016). Effects of water stress and light intensity on chlorophyll fluorescence parameters and pigments of aloe vera l. Plant Physiol. Biochem. 106, 141–148. doi: 10.1016/j.plaphy.2016.04.046 27161580

[B20] Hodges D. M. Delong J. M. Forney C. F. Prange R. K. (1999). Improving the thiobarbituric acid-reactive-substances assay for estimating lipid peroxidation in plant tissues containing anthocyanin and other interfering compounds. Planta 207, 604–611. doi: 10.1007/s004250050524 28456836

[B21] Huang C. Gao Y. Qin A. Z. Liu Z. G. Zhao B. Ning D. F.. (2022). Effects of waterlogging at different stages and durations on maize growth and grain yields. Agric. Water Manage. 261. doi: 10.1016/j.agwat.2021.107334

[B22] Jbir-Koubaa R. Charfeddine S. Ellouz W. Saidi M. N. Drira N. Gargouri-Bouzid R.. (2015). Investigation of the response to salinity and to oxidative stress of interspecific potato somatic hybrids grown in a greenhouse. Plant Cell Tissue Organ Culture 120, 933–947. doi: 10.1007/s11240-014-0648-4

[B23] Jiang Y. P. Ding X. T. Wang J. Y. Zou J. Nie W. F. (2021). Decreased low-light regulates plant morphogenesis through the manipulation of hormone biosynthesis in solanum lycopersicum. Environ. Exp. Bot. 52 (355), 341–9. doi: 10.1016/j.envexpbot.2021.104409

[B24] Jiang Y. Huang B. (2001). Effects of calcium on antioxidant activities and water relations associated with heat tolerance in two cool-season grasses. J. Exp. Bot. 52 (355), 341–349. doi: 10.1093/jexbot/52.355.341 11283179

[B25] Khan A. L. Hussain J. Al-Harrasi A. Al-Rawahi A. Lee I. J. (2015). Endophytic fungi: resource for gibberellins and crop abiotic stress resistance. Crit. Rev. Biotechnol. 35, 62–74. doi: 10.3109/07388551.2013.800018 23984800

[B26] Lee S.-H. Ahsan N. Lee K.-W. Kim D.-H. Lee D.-G. Kwak S.-S.. (2007). Simultaneous overexpression of both CuZn superoxide dismutase and ascorbate peroxidase in transgenic tall fescue plants confers increased tolerance to a wide range of abiotic stresses. J. Plant Physiol. 164, 1626–1638. doi: 10.1016/j.jplph.2007.01.003 17360071

[B27] Li J. K. Essemine J. Shang C. Zhang H. L. Zhu X. C. Yu J. L.. (2020). Combined proteomics and metabolism analysis unravels prominent roles of antioxidant system in the prevention of alfalfa (*Medicago sativa* l.) against salt stress. Int. J. Mol. Sci. 21 (3), 909. doi: 10.3390/ijms21030909 32019165 PMC7037825

[B28] Lichtenthaler H. K. Wellburn A. R. (1983). Determinations of total carotenoids and chlorophylls a and b of leaf extracts in different solvents. Analysis 11, 591–592. doi: 10.1007/s004250050524

[B29] Liu Y. X. Pan T. Tang Y. Y. Zhuang Y. Liu Z. J. Li P. H.. (2020). Proteomic analysis of rice subjected to low light stress and overexpression of OsGAPB increases the stress tolerance. Rice 13. doi: 10.1186/s12284-020-00390-8 PMC7266901 32488648

[B30] Magome H. Yamaguchi S. Hanada A. Kamiya Y. Oda K. (2004). Dwarf and delayed-flowering 1, a novel arabidopsis mutant deficient in gibberellin biosynthesis because of overexpression of a putative AP2 transcription factor. Plant J. Cell Mol. Biol. 37, 720–729. doi: 10.1111/j.1365-313X.2003.01998.x 14871311

[B31] Mcatee P. Karim S. Schaffer R. David K. (2013). A dynamic interplay between phytohormones is required for fruit development, maturation, and ripening. Front. Plant Sci. 4. doi: 10.3389/fpls.2013.00079 PMC3628358 23616786

[B32] Mittler R. (2002). Oxidative stress, antioxidants and stress tolerance. Trends Plant Sci. 7, 405–410. doi: 10.1016/s1360-1385(02)02312-9 12234732

[B33] Ni B. R. Bradford K. J. (1993). Germination and dormancy of abscisic acid- and gibberellin-deficient mutant tomato (*Lycopersicon esculentum*) seeds (Sensitivity of germination to abscisic acid, gibberellin, and water potential). Plant Physiol. 101, 607–617. doi: 10.1104/pp.101.2.607 12231716 PMC160610

[B34] Qian C. J. Zhang W. Zhong X. M. Li F. H. Shi Z. S. (2017). Comparative studies on the photosynthetic characteristics of two maize (Zea mays l.) near-isogenic lines differing in their susceptibility to low light intensity. Emirates J. Food Agric. 29, 300–311. doi: 10.9755/ejfa.2016-07-839

[B35] Qu M. N. Zheng G. Y. Hamdani S. Essemine J. Song Q. F. Wang H. R.. (2017). Leaf photosynthetic parameters related to biomass accumulation in a global rice diversity survey. Plant Physiol. 175, 248–258. doi: 10.1104/pp.17.00332 28739819 PMC5580745

[B36] Ramel F. Sulmon C. Bogard M. Couee I. Gouesbet G. (2009). Differential patterns of reactive oxygen species and antioxidative mechanisms during atrazine injury and sucrose-induced tolerance in arabidopsis thaliana plantlets. BMC Plant Biol. 9. doi: 10.1186/1471-2229-9-28 PMC2661893 19284649

[B37] Rizza A. Walia A. Lanquar V. Frommer W. B. Jones A. M. (2017). *In vivo* gibberellin gradients visualized in rapidly elongating tissues. Nat. Plants 3, 803–813. doi: 10.1038/s41477-017-0021-9 28970478

[B38] Rood S. B. Mandel R. Pharis R. P. (1989). Endogenous gibberellins and shoot growth and development in brassica napus. Plant Physiol. 89, 269–273. doi: 10.1104/pp.89.1.269 16666524 PMC1055830

[B39] Sauret-Gueto S. Calder G. Harberd N. P. (2012). Transient gibberellin application promotes arabidopsis thaliana hypocotyl cell elongation without maintaining transverse orientation of microtubules on the outer tangential wall of epidermal cells. Plant J. 69, 628–639. doi: 10.1111/j.1365-313X.2011.04817.x 21985616

[B40] Slattery R. A. Ort D. R. (2021). Perspectives on improving light distribution and light use efficiency in crop canopies. Plant Physiol. 185, 34–48. doi: 10.1093/plphys/kiaa006 33631812 PMC8133579

[B41] Smith E. L. (1938). LIMITING FACTORS IN PHOTOSYNTHESIS: LIGHT AND CARBON DIOXIDE. J. Gen. Physiol. 22, 21–35. doi: 10.1085/jgp.22.1.21 19873090 PMC2213731

[B42] Verma V. Ravindran P. Kumar P. P. (2016). Plant hormone-mediated regulation of stress responses. BMC Plant Biol. 16. doi: 10.1186/s12870-016-0771-y PMC4831116 27079791

[B43] Vicente M. R. S. Plasencia J. (2011). Salicylic acid beyond defence: its role in plant growth and development. J. Exp. Bot. 62, 3321–3338. doi: 10.1093/jxb/err031 21357767

[B44] Wang H. P. Pan J. J. Li Y. Lou D. J. Hu Y. R. Yu D. Q. (2016). The DELLA-CONSTANS transcription factor cascade integrates gibberellic acid and photoperiod signaling to regulate flowering. Plant Physiol. 172, 479–488. doi: 10.1104/pp.16.00891 27406167 PMC5074646

[B45] Wang J. Wang D. Zhu M. Li F. (2021). Exogenous 6-benzyladenine improves waterlogging tolerance in maize seedlings by mitigating oxidative stress and upregulating the ascorbate-glutathione cycle. Front. Plant Sci. 12. doi: 10.3389/fpls.2021.680376 PMC8446516 34539688

[B46] Wang X. Xu C. Cang J. Zeng Y. Yu J. Liu L.. (2015). Effects of exogenous GA(3) on wheat cold tolerance. J. Agric. Sci. Technol. 17 (4), 921–934.

[B47] Wegrzyn A. Mazur R. (2020). Regulatory mechanisms of photosynthesis light reactions in higher plants. Postepy biochemii 66, 134–142. doi: 10.18388/pb.2020_325 32700507

[B48] Wiszniewska A. Muszynska E. Hanus-Fajerska E. Dziurka K. Dziurka M. (2018). Evaluation of the protective role of exogenous growth regulators against Ni toxicity in woody shrub Daphne jasminea. Planta 248, 1365–1381. doi: 10.1007/s00425-018-2979-6 30116887 PMC6244662

[B49] Xia X.-J. Wang Y.-J. Zhou Y.-H. Tao Y. Mao W.-H.. (2009). Reactive oxygen species are involved in brassinosteroid-induced stress tolerance in cucumber. Plant Physiol. 150, 801–814. doi: 10.1104/pp.109.138230 19386805 PMC2689980

[B50] Xu Z. Zhang J. Wang X. Essemine J. Jin J. Qu M.. (2023). Cold-induced inhibition of photosynthesis-related genes integrated by a TOP6 complex in rice mesophyll cells. Nucleic Acids Res. doi: 10.1093/nar/gkac1275 PMC9976896 36660855

[B51] Xue J. Li T. Wang S. Xue Y. Liu X.. (2019). Defoliation and gibberellin synergistically induce tree peony flowering with non-structural carbohydrates as intermedia. J. Plant Physiol. 233, 31–41. doi: 10.1016/j.jplph.2018.12.004 30580057

[B52] Yamaguchi N. Winter C. M. Wu M. F. Kanno Y. Yamaguchi A. Seo M.. (2014). Gibberellin acts positively then negatively to control onset of flower formation in arabidopsis. Science 344, 638–641. doi: 10.1126/science.1250498 24812402

[B53] Yamori W. (2016). Photosynthetic response to fluctuating environments and photoprotective strategies under abiotic stress. J. Plant Res. 129, 379–395. doi: 10.1007/s10265-016-0816-1 27023791

[B54] Yang Y. S. Guo X. X. Hou P. Xue J. Liu G. Z. Liu W. M.. (2020). Quantitative effects of solar radiation on maize lodging resistance mechanical properties. Field Crops Res. 255. doi: 10.1016/j.fcr.2020.107906

[B55] Yu K. Wang Y. Wei J. Ma Q. Yu D. Li J. R. (2009). Improving rhizome yield and quality of Paris polyphylla through gibberellic acid-induced retardation of senescence of aerial parts. Plant Signaling Behav. 4, 413–415. doi: 10.4161/psb.4.5.8268 PMC2676751 19816118

[B56] Zahra N. Al Hinai M. S. Hafeez M. B. Rehman A. Wahid A. Siddique K. H. M.. (2022). Regulation of photosynthesis under salt stress and associated tolerance mechanisms. Plant Physiol. Biochem. PPB 178, 55–69. doi: 10.1016/j.plaphy.2022.03.003 35276596

[B57] Zentella R. Zhang Z.-L. Park M. Thomas S. G. Endo A. Murase K.. (2007). Global analysis of della direct targets in early gibberellin signaling in arabidopsis. Plant Cell 19, 3037–3057. doi: 10.1105/tpc.107.054999 17933900 PMC2174696

[B58] Zhang H. H. Wang Y. Li X. He G. Q. Che Y. H. Teng Z. Y.. (2020). Chlorophyll synthesis and the photoprotective mechanism in leaves of mulberry (Mores alba l.) seedlings under NaCl and NaHCO3 stress revealed by TMT-based proteomics analyses. Ecotoxicology Environ. Saf. 190. doi: 10.1016/j.ecoenv.2020.110164 31927191

[B59] Zhong S. W. Shi H. Xue C. Wang L. Xi Y. P. Li J. G.. (2012). A molecular framework of light-controlled phytohormone action in arabidopsis. Curr. Biol. 22, 1530–1535. doi: 10.1016/j.cub.2012.06.039 22818915 PMC4437768

[B60] Zhou S. He L. Lin W. Su Y. Liu Q. Qu M.. (2022). Integrative analysis of transcriptome and metabolism reveals potential roles of carbon fixation and photorespiratory metabolism in response to drought in shanlan upland rice. BMC Genomics 23, 862. doi: 10.1186/s12864-022-09094-3 36585635 PMC9805275

[B61] Zivcak M. Brestic M. Kalaji H. M. Govindjee (2014). Photosynthetic responses of sun- and shade-grown barley leaves to high light: is the lower PSII connectivity in shade leaves associated with protection against excess of light? Photosynthesis Res. 119, 339–354. doi: 10.1007/s11120-014-9969-8 PMC3923118 24445618

