# Supplementary material for: Effect of gibberellic acid on photosynthesis and oxidative stress response in maize under weak light conditions
Source: Front Plant Sci. 2023 Feb 16;14:1128780. doi: 10.3389/fpls.2023.1128780 (PMC9978513; doi:10.3389/fpls.2023.1128780)
Supplement: Supplementary file 1 [file DataSheet_1.pdf]

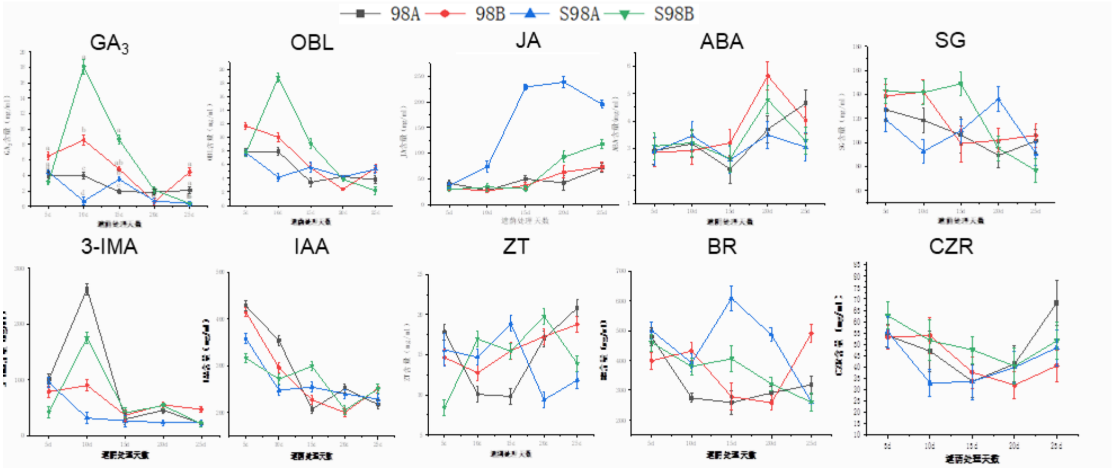

The experiment was conducted to study the changes of hormone contents of SN98A and SN98B before and after shade, in which 98A and SN98B were SN98A and SN98B without shade,S98A and S98B were SN98A and SN98B after 38% shade. The hormone GA_3, OBL was higher than SN98A after shade, and GA_3, OBL had the effect of promoting seed setting, so GA_3 was selected as the spraying hormone. OBL is not applicable in actual production, so it is not chosen.
